# A Factor-Graph-Based Approach to Vehicle Sideslip Angle Estimation

**DOI:** 10.3390/s21165409

**Published:** 2021-08-10

**Authors:** Antonio Leanza, Giulio Reina, José-Luis Blanco-Claraco

**Affiliations:** 1 Department of Mechanics, Mathematics, and Management, Polytechnic of Bari, via Orabona 4, 70126 Bari, Italy; antonio.leanza@unisalento.it (A.L.); giulio.reina@poliba.it (G.R.); 2 Department of Engineering, Campus de Excelencia Internacional Agroalimentario, University of Almería, ceiA3, 04120 Almería, Spain

**Keywords:** vehicle dynamics estimation, sideslip angle estimation, factor graph, graphical models, Kalman filtering

## Abstract

Sideslip angle is an important variable for understanding and monitoring vehicle dynamics, but there is currently no inexpensive method for its direct measurement. Therefore, it is typically estimated from proprioceptive sensors onboard using filtering methods from the family of the Kalman filter. As a novel alternative, this work proposes modeling the problem directly as a graphical model (factor graph), which can then be optimized using a variety of methods, such as whole-dataset batch optimization for offline processing or fixed-lag smoothing for on-line operation. Experimental results on real vehicle datasets validate the proposal, demonstrating a good agreement between estimated and actual sideslip angle, showing similar performance to state-of-the-art methods but with a greater potential for future extensions due to the more flexible mathematical framework. An open-source implementation of the proposed framework has been made available online.

## 1. Introduction

A large body of research work has accumulated during the past three decades regarding sideslip estimation, which is a fundamental feature of vehicle dynamics [1]. Despite its central role, the direct measurement of sideslip angle over time is often impractical during vehicle motion, for both technical and economic reasons. For instance, a cheap but few practical solution is to use measures coming from the Global Positioning System (GPS) that can provide the position of the receiver without any numerical integration and then derive the velocity of the vehicle using Doppler measurements [2]. Nevertheless, GPS receivers have issues such as temporary signal unavailability due to signal blocking by trees, buildings, or urban canyons, as well as (typically) a much lower measuring rate than other onboard sensors involved in vehicle dynamics control such as accelerometers or gyroscopes. On the other hand, direct measurement of the vehicle sideslip angle can be achieved by high-precision optical sensors, but they are sophisticated, still in an early research and development (R&D) stage, and expensive for production vehicles [3].

For these reasons, sideslip angle estimation continues to pose a significant challenge in vehicle dynamics research, attracting noticeable interest in both the academic and industrial worlds [4,5,6]. Several methods have been developed and described throughout the scientific literature which make use of different models and estimators. The most common approach relies on model-based observers [7,8,9,10,11], which make use of a vehicle reference model for state and parameter estimators. Different levels of sophistication can be obtained in order to achieve a phenomenon description that is as accurate as possible. One of the most common combinations that can be found in the literature is the bicycle (single-track) model as an observer for a Kalman filter (KF). This arrangement allows the estimation of states and parameters at the same time. For instance, in [12,13] a linear bicycle model is used in combination with an extended Kalman filter (EKF) for the estimation of the sideslip angle and vehicle parameters at the same time. The former estimates lateral velocity and vehicle mass by also correcting the bias of the gyroscope, while the latter estimates the front and rear cornering stiffness and the sideslip angle directly as a dynamic state. In [14], the same linear model is exploited but using a cubature Kalman filter (CKF) as the estimator for obtaining the sideslip angle and other states from the input noisy measurements (i.e., accelerations, vehicle longitudinal velocity, etc.). In [15], a combined approach between kinematic and dynamic models is carried out, since the kinematic model performs well during transient maneuvers but fails in steady-state conditions. Therefore, the information provided by the kinematic formulation is exploited to update the single-track model parameters (i.e., the tire cornering stiffness), while the dynamic state observer is used in the nearly quasi-state condition. The steady-state or transient conditions are discriminated via a fuzzy-logic procedure. Furthermore, the bicycle model can also be coupled with nonlinear tire models, as in [16], where the authors attempt to estimate the sideslip angle of a heavy-duty vehicle by using a rational tire model and an EKF as the estimator. The study showed good estimation performance, but at the cost of an overparametrized and complex model. On the other hand, four-contact models provide a better description of vehicle dynamics, but obviously at the cost of a greater number of parameters and an increase in the complexity of the systems. For instance, the authors in [17] use an EKF applied to a four-contact vehicle model with a Dugoff tire model in order to estimate the sideslip angle and the tire/road forces. The authors in [18] again use a four-contact model, but with a semiphysical nonlinear tire model called “Unitire”, and apply a reduced-order sliding mode observer (SMO), evaluating the performance of the proposed method by means of simulation and experiments. In [19] the Magic Formula (or Pacejka tire model [20]) is exploited, where a preliminary filtering on vertical forces is performed via linear KF in order to estimate the roll angle and then an EKF is applied to the four-contact vehicle model to achieve the sideslip angle. Again, the Magic Formula with a four-contact vehicle model is used in [21], where a dual estimation scheme was adopted: the sideslip angle and tire/road forces by means of a dual unscented Kalman filter (UKF) algorithm and the Pacejka tire parameters by solving a nonlinear least squares (LS) problem. However, the sophistication of these models might be unpractical in real applications. To overcome this issue, some authors (e.g., [22]) rely on direct causality equations without the need of any explicit tire model. In the cited research, different estimators are developed, such as the standard EKF, the CKF, and particle filtering (PF), and the results are evaluated using a vehicle model with 14 degrees of freedom (DOFs) which performs standard maneuvers. Authors in [23] perform vehicle sideslip angle and road bank angle estimation via a simple algebraic relationship in real time, based on two online parameter identification techniques, combining a single-track model with roll and tire slip models and force due to bank angle. Moreover, with the use of a lateral G sensor signal and by including road bank angle effect, the front and rear cornering stiffness and vehicle sideslip angle are identified, in the absence of any a priori knowledge of the road bank angle. In order to completely overcome the need for a vehicle model of any kind and its related complex set of parameters, different approaches based on artificial neural networks (ANNs) [24,25,26,27] are now widely investigated, since they are suitable for modeling complex systems using their ability to identify relationships from input–output data.

In this paper, we present a novel estimation technique grounded in factor graphs (FGs) theory. To the best of our knowledge, this is the first time an FG-based observer is proposed in the automotive field. An FG is one of the possible types of probabilistic graphical models that can be used to describe the structure of an estimation problem [28,29]. Factor graphs are bipartite graphs comprising two kinds of nodes: variable and factor nodes. Variable nodes represent the unknown data to be estimated, while factors represent cost functions to be minimized, modeling relationships between the variables. Each factor node is connected to only those variables that appear in its cost function; this allows an FG to explicitly model the key property of how sparse a problem is, depending on the connection pattern between its variables. As shown below, by applying this estimation approach to the classical linear bicycle model, good estimation accuracy can be achieved even in the nonlinear regions of the tire behavior compared to the celebrated Kalman filtering, while preserving the inherent simplicity of the linear model and the use of few parameters (i.e., the front and rear cornering stiffness).

The paper is organized as follows. In Section 2 the equations related to the linear bicycle model are recalled and reorganized in order to directly obtain the sideslip angle. Section 3 introduces the estimation problem with the use of FGs, applied to the case at hand with details on its implementation. Results obtained with the proposed method are evaluated by means of real data gathered in the Stanford database [30] in Section 4. Finally, conclusions are drawn in Section 5.

## 2. Vehicle Dynamic Model

**Figure 1:**
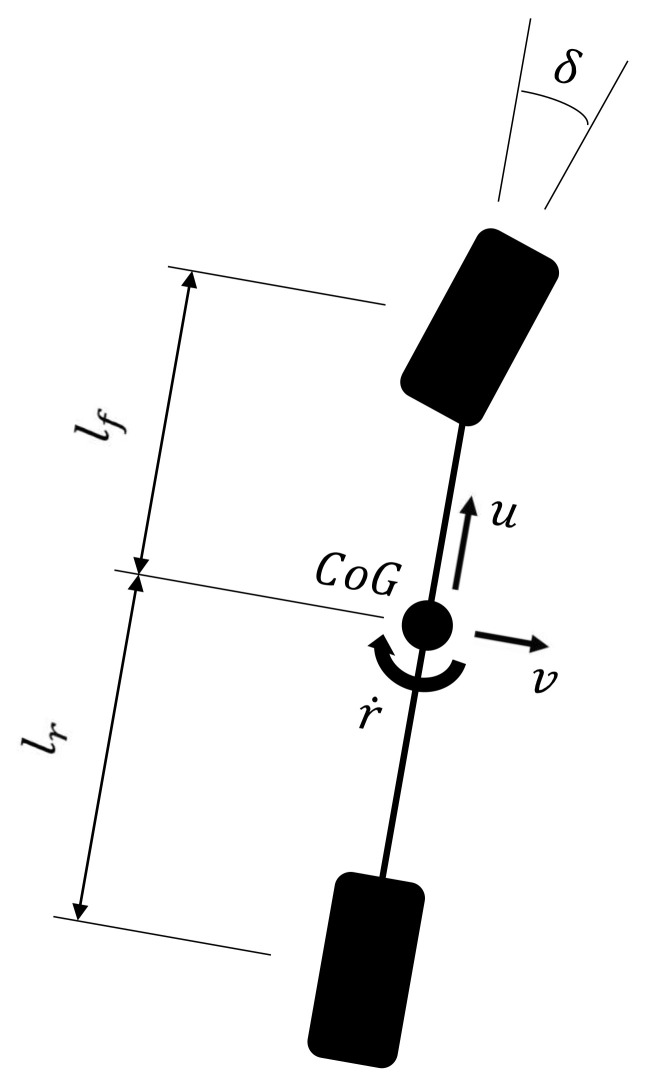
Single-track model.

Lateral dynamics represents a basic challenge in vehicle behavior, because lateral velocity is usually not directly measurable for practical and/or economic reasons. Nonetheless, it represents fundamental information, since it influences the entire vehicle dynamics, especially the direction of motion. In fact, the total velocity of the vehicle center of gravity (CoG) is the vectorial sum of the longitudinal and lateral velocities *u* and *v*, respectively. Figure 1 shows the model used in this paper for dealing with the vehicle’s lateral behavior. It is known as a “bicycle” or “single-track” model, and it is based on the following simplifications [31]: equal internal and external dynamics so that the tires of the same axle can be fused, leading to the model’s bicycle-like appearance; linear range of the tires, thus lateral forces are a linear function of slip angles by means of a specific coefficient named cornering stiffness; rear-wheel drive; negligible motion resistance; small-angle approximation to preserve the linearity of the model; constant longitudinal velocity *u*, which is guaranteed within a small time-step Δt. This model is characterized by two degrees of freedom (DoFs)—namely, the vehicle lateral velocity *v* and the yaw rate *r*. Hence, two equations of motion are sufficient to fully describe its behavior over time:

(1) $$\begin{array}{cc} & \dot{v}=-\left(\frac{C_{f}+C_{r}}{mu}\right)v-\left(\frac{C_{f}l_{f}-C_{r}l_{r}}{mu}+u\right)r+\frac{C_{f}\delta }{m}\\ & \dot{r}=-\left(\frac{C_{f}l_{f}-C_{r}l_{r}}{J_{z}u}\right)v-\left(\frac{C_{f}l_{f}^{2}+C_{r}l_{r}^{2}}{J_{z}u}\right)+\frac{C_{f}l_{f}\delta }{J_{z}} \end{array}$$

where *m* is the vehicle mass, $J_{z}$ is the moment of inertia with respect to the yaw axis, $C_{f}$ and $C_{r}$ are the front and rear cornering stiffness, respectively, $l_{f}$ and $l_{r}$ are respectively the distance of the CoG from the front and rear axle, and δ is the front steering angle.

Usually, the sideslip angle β is of interest in the study of lateral dynamics, in place of the lateral velocity *v*; therefore, one can consider as DoFs β and *r* in place of the couple *v* and *r*. From Figure 1, the relation between *v* and β is the following:

(2) $$\beta =\arctan\left(\frac{v}{u}\right)\approx \frac{v}{u}$$

where the approximation comes from the assumption of small angles, as stated above. Therefore,

(3) v=βuandv˙=β˙u+βu˙0≈β˙u,

considering the constancy of *u* within each time-step Δt. The Equation (1) become:

(4) $$\begin{array}{cc} & \dot{\beta }=-\left(\frac{C_{f}+C_{r}}{mu}\right)\beta -\left(\frac{C_{f}l_{f}-C_{r}l_{r}}{mu^{2}}+1\right)r+\frac{C_{f}\delta }{mu}\\ & \dot{r}=-\left(\frac{C_{f}l_{f}-C_{r}l_{r}}{J_{z}}\right)\beta -\left(\frac{C_{f}l_{f}^{2}+C_{r}l_{r}^{2}}{J_{z}u}\right)+\frac{C_{f}l_{f}\delta }{J_{z}} \end{array}$$

By considering Equation (4), β and *r* are variables of the vehicle lateral dynamics and the other terms are considered to be known parameters.

It is worth noting that the yaw rate *r* can be directly measured by means of a vertical gyroscope, while the sideslip angle β usually has to be estimated. Although β can also be measured via GPS and Equation (2), this type of measurement is often unstable and unreliable due to the presence of shady areas of the GPS signal, such as tunnels or mountains. This leads to the need to indirectly estimate the sideslip angle. To achieve this estimation, the lateral acceleration $a_{y}$ is considered as a further measurement. Both $a_{y}$ and *r* are generally already available onboard common vehicles via the ESP system. Therefore, the following set of measurements is here considered:

(5) $$\begin{array}{cc} & \dot{\varphi }=r\\ & a_{y}=\dot{v}+ur=u\left(\dot{\beta }+r\right) \end{array}$$

As a matter of practicality, Equations (4) and (5) in their continuous-time form need to be converted into the following discrete-time representations, obtained from the forward Euler integration with time step $\Delta t=t_{k}-t_{k-1}$:

(6) $$\begin{array}{ccc} \beta_{k} & = & \beta_{k-1}+\Delta t\left[-\frac{C_{f}+C_{r}}{mu_{k-1}}\beta_{k-1}-\left(\frac{C_{f}l_{f}-C_{r}l_{r}}{mu_{k-1}^{2}}+1\right)r_{k-1}+\frac{C_{f}\delta_{k-1}}{mu_{k-1}}\right] \end{array}$$

(7) $$\begin{array}{ccc} r_{k} & = & r_{k-1}+\Delta t\left(-\frac{C_{f}l_{f}-C_{r}l_{r}}{Jz}\beta_{k-1}-\frac{C_{f}l_{f}^{2}+C_{r}l_{r}^{2}}{Jzu_{k-1}}r_{k-1}+\frac{C_{f}l_{f}\delta_{k-1}}{Jz}\right) \end{array}$$

(8) $$\begin{array}{ccc} {\dot{\varphi }}_{k} & = & r_{k}a_{yk}=-\frac{C_{f}+C_{r}}{m}\beta_{k}-\frac{C_{f}l_{f}-C_{r}l_{r}}{mu_{k}}r_{k}+\frac{C_{k}\delta_{k}}{m} \end{array}$$

## 3. Factor Graph for Vehicle Lateral Dynamics

### 3.1. The Estimation Problem

The discrete-time Equation (6) refer to the vehicle system and to the measurements gathered for a correct estimate of the DoFs, in particular for the estimation of β, since *r* is directly measured. The most common method followed throughout the literature relies on a state-space representation of the above equations, writing two compacted matrix equations: one for the propagation of the model over time and one relating to the measures, both as a function of suitable state variables. Therefore, a recursive routine, often based on Kalman filtering, is performed to achieve the correct estimation of the unknown state variables [22].

**Figure 2:**
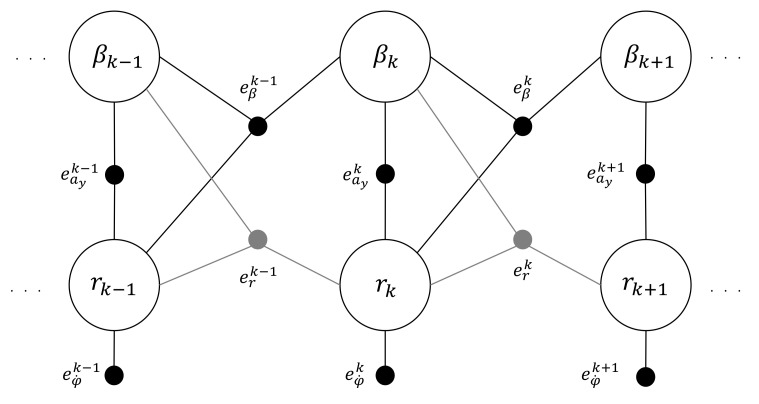
Factor graph (FG) relative to the linear bicycle model.

In this paper, an alternative approach is suggested for the estimation of the unknown variables, without passing through the state-space form—namely, the factor graphs (FGs), a method belonging to the family of probabilistic graphical models. The objective is to minimize given cost functions in each time step. The FG relative to the problem at hand is displayed in Figure 2 for a generic time-step *k*. Each factor (filled circle in the figure) is connected with the unknown variables involved in the cost function that the factor minimizes. The cost functions come directly from Equation (8):

(9) $$\begin{array}{ccc} e_{\beta }^{k-1} & = & \beta_{k}-\beta_{k-1}-\Delta t\left[-\frac{C_{f}+C_{r}}{mu_{k-1}}\beta_{k-1}-\left(\frac{C_{f}l_{f}-C_{r}l_{r}}{mu_{k-1}^{2}}+1\right)r_{k-1}+\frac{C_{f}\delta_{k-1}}{mu_{k-1}}\right] \end{array}$$

(10) $$\begin{array}{ccc} e_{r}^{k-1} & = & r_{k}-r_{k-1}-\Delta t\left(-\frac{C_{f}l_{f}-C_{r}l_{r}}{Jz}\beta_{k-1}-\frac{C_{f}l_{f}^{2}+C_{r}l_{r}^{2}}{Jzu_{k-1}}r_{k-1}+\frac{C_{f}l_{f}\delta_{k-1}}{Jz}\right) \end{array}$$

(11) $$\begin{array}{ccc} e_{\dot{\varphi }}^{k} & = & \dot{\varphi }-r_{k} \end{array}$$

(12) $$\begin{array}{ccc} e_{a_{y}}^{k} & = & a_{yk}+\frac{C_{f}+C_{r}}{m}\beta_{k}+\frac{C_{f}l_{f}-C_{r}l_{r}}{mu_{k}}r_{k}-\frac{C_{f}\delta_{k}}{m} \end{array}$$

The cost functions $e_{\beta }^{k-1}$ and $e_{r}^{k-1}$ are handled, respectively, by the black and gray ternary factors (Figure 2). On the other hand, the unary and binary factors are referred to the measurements: the unary factor minimizes the difference between the actual yaw rate $\dot{\varphi }$ and the associated unknown *r*, whilst the binary factor minimizes the difference between the actual lateral acceleration $a_{y}$ and the estimated one.

In principle, the value of the error functions (12) has to be equal to zero, but actually an uncertainty is associated to each factor due to the stochastic nature of the estimation problems. First, measurements are collected through the use of sensors with inherent precision and accuracy. The uncertainties associated to the model include mathematical approximations and uncertainties of the inputs *u* and δ.

Without loss of generality, with the assumption of a zero-mean Gaussian probability density function (pdf) associated to the uncertainty in each factor, the minimization problem can be reduced to a linear least squares problem. Since the unknown variables $X_{k}$ to minimize at the *k*-th instant are ${\left[\beta_{k}\;r_{k}\right]}^{T}$, Equation (12) can be written in compact matrix form as:

(13) $${\left[\begin{array}{c} e_{\beta }\\ e_{r}\\ e_{\dot{\varphi }}\\ e_{a_{y}} \end{array}\right]}_{k}=H_{k}X_{k}+C_{k}h_{k}$$

where

$$\begin{array}{cc} & H_{k}=\left[\begin{array}{ccccccc} -\left(1-\Delta t\frac{C_{f}+C_{r}}{mu_{k-1}}\right) & & \Delta t\left(\frac{C_{f}l_{f}-C_{r}l_{r}}{mu_{k-1}^{2}}+1\right) & & 1 & & 0\\ \Delta t\frac{C_{f}l_{f}-C_{r}l_{r}}{J_{z}} & & -\left(1-\Delta t\frac{C_{f}l_{f}^{2}+C_{r}l_{r}^{2}}{Jzu_{k-1}}\right) & & 0 & & 1\\ 0 & & 0 & & 0 & & -1\\ 0 & & 0 & & \frac{C_{f}+C_{r}}{m} & & \frac{C_{f}l_{f}-C_{r}l_{r}}{mu_{k}} \end{array}\right]\\ & C_{k}=\mathrm{diag}\left(\frac{C_{f}}{mu_{k-1}}\;\frac{C_{f}l_{f}}{J_{z}}\;0\;-\frac{C_{f}}{m}\right)\\ & h_{k}={\left[\delta_{k-1}\;\delta_{k-1}\;0\;\delta_{k}\right]}^{T} \end{array}$$

By defining the *state update vector* as $\Delta_{k}:=X_{k}-X_{k-1}$, the next equation holds:

(14) $$H_{k}X_{k}=H_{k}\Delta_{k}+H_{k}X_{k-1}$$

The target is the estimation of the state update vector $\Delta_{k}$ via least squares. It is important to remark that the problem at hand is linear; therefore, the following weighted least squares (WLS) problem is set:

(15) $$\hat{\Delta }=\underset{\Delta }{\mathrm{argmin}}\sum_{k}∥H_{k}\Delta_{k}-\left(z_{k}-H_{k}X_{k-1}-C_{k}h_{k}\right){∥}_{Q_{k}}^{2}$$

where $z_{k}$ is the *k*-th vector of measures, $Q_{k}$ is the error covariance matrix and the parenthesis $\left(z_{k}-H_{k}X_{k-1}-C_{k}h_{k}\right)$ is the *prediction error*. The argument of the sum is the Mahalanobis norm:

(16) $${\left[Q_{k}^{-\frac{1}{2}}\left[H_{k}\Delta_{k}-\left(z_{k}-H_{k}X_{k-1}-C_{k}h_{k}\right)\right]\right]}^{T}\left[Q_{k}^{-\frac{1}{2}}\left[H_{k}\Delta_{k}-\left(z_{k}-H_{k}X_{k-1}-C_{k}h_{k}\right)\right]\right]$$

and from the following replacements:

(17) $$\begin{array}{ccc} Q_{k}^{-\frac{1}{2}}H_{k} & = & A_{k} \end{array}$$

(18) $$\begin{array}{ccc} Q_{k}^{-\frac{1}{2}}\left(z_{k}-H_{k}X_{k-1}-C_{k}h_{k}\right) & = & b_{k} \end{array}$$

one obtains the following simple least squares problem:

(19) $$\hat{\Delta }=\underset{\Delta }{\mathrm{argmin}}∥A\Delta -b{∥}_{2}^{2}={\left(\;A^{T}A\right)}^{-1}A^{T}b$$

where A is a large matrix collecting all matrices $A_{k}$. From the calculation of the state update vector: ${\hat{X}}_{k}={\hat{X}}_{k-1}+{\hat{\Delta }}_{k}$, where A, b, and Δ grow over time.

### 3.2. Implementation

**Figure 3:**
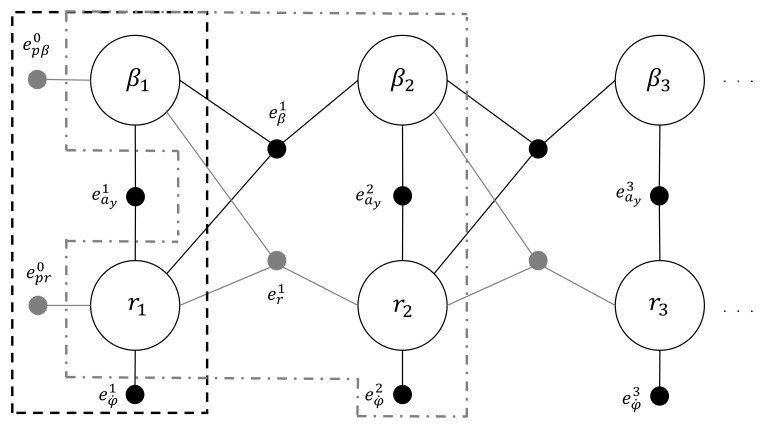
FG relative to the first three steps of the estimation problem: the first step with priors is shown in the black dashed window, and the generic step is shown in the gray dashed-dotted window, with the involved factors.

Figure 3 displays the first three steps for the estimation problem at hand. The first step, surrounded by the closed dashed black line, includes two prior factors (the unitary gray ones) in place of the dynamic factors. The prior factors minimize the difference between the first estimates and the initial guess of the unknown variables, enforcing known initial conditions; therefore, a reliable initial guess is assumed, with values very close to the correct ones. Their error functions are:

(20) $$\begin{array}{ccc} e_{p\beta }^{0} & = & \beta_{1}-\beta_{0} \end{array}$$

(21) $$\begin{array}{ccc} e_{pr}^{0} & = & r_{1}-r_{0} \end{array}$$

The other steps follow the logic of the second step, contained in the closed dashed-dotted gray line. This line encloses four unknown variables and four factors: two belonging to the dynamic model and two for the measures. By looking at the first dynamic step enclosed in the gray line, the dynamic model is managed by the ternary factors $e_{\beta }^{1}$ and $e_{r}^{1}$ for the estimation of the sideslip angle and the yaw rate, respectively, while factors $e_{a_{y}}^{2}$ and $e_{\dot{\varphi }}^{2}$ introduce new measurements and minimize the difference between what is effectively measured and the what is expected from the model.

In order to understand how to move from the graphical representation of factors to the minimization problem, the A matrix and vectors b and Δ are shown for the first three steps of Figure 3.

In Table 1 matrix A and vector b are reported, and the Δ vector for the first three steps is that in Equation (22).

(22) $$\left[\begin{array}{c} \Delta_{1}\\ \Delta_{2}\\ \Delta_{3}\\ \Delta_{4}\\ \Delta_{5}\\ \Delta_{6} \end{array}\right]=\left[\begin{array}{c} \beta_{1}-\beta_{0}\\ r_{1}-r_{0}\\ \beta_{2}-\beta_{1}\\ r_{2}-r_{1}\\ \beta_{3}-\beta_{2}\\ r_{3}-r_{2} \end{array}\right]$$

Matrix A in Table 1 is composed of blocks, each one corresponding to a single time-step of the FG, as shown in Figure 3. The first block corresponds to the black dashed window containing the two priors, two measures, and the first two unknown variables $\beta_{1}$ and $r_{1}$. Hence, the whitened matrix $A_{1}$ is a 4×2 matrix, where each row is associated to a specific factor of Figure 3. The whitened matrix $A_{2}$ is composed of a square matrix containing the first and second blocks together, and so forth. Vectors b and Δ grow accordingly. It is worth noting that the resulting matrix is a sparse matrix, with elements concentrated near the diagonal. This characteristic can be exploited in the minimization problem of Equation (19). For further details on the elements of matrix A and vector b in Table 1, the interested reader can refer to the Appendix A.

Matrix $Q_{k}$ collects the uncertainty associated to each factor. Therefore, $Q_{k}^{-\frac{1}{2}}$ contains the weights of the factors, representing the tuning parameters of this estimation problem. The greater the confidence given to a factor, the higher the values associated with its weight and the lower the corresponding standard deviation. In this case, by looking at Equation (12) it is clear that the factor associated to the yaw rate measurement has a large weight, being precisely measured; therefore, the standard deviation associated to the error of Equation (11) is very small. In this work, a value of $\sigma_{\dot{\varphi }}=10^{-8}$ rad/s is assumed. Instead, the error associated to the Equation (12) relative to the lateral acceleration measurement is taken to be larger because a linear model is compared with the actual lateral acceleration; therefore, a smaller weight is associated to that factor by assuming a $\sigma_{a_{y}}=10^{-2}$ $m/s^{2}$. Regarding the error relative to the model equations, a high weight is associated to the corresponding factors for the well-known reliability of the model, with standard deviations equal to $\sigma_{\beta }=10^{-5}$ rad and $\sigma_{r}=10^{-4}$ rad/s for the sideslip angle model and yaw rate model, respectively.

During vehicle motion, a large amount of data can be collected. Two estimator implementations are explored: performing a batch estimation on the whole dataset, and a fixed-lag-smoother. The latter considers a sliding window, which contains a fixed number of samples *M*. Hence, the minimization is achieved on these samples and the most reliable estimate is retained as a set of priors for the next estimation. The length of the sliding window is a tuning parameter. In this work, a window length of M=5 samples is heuristically found to be a a good trade-off between computational speed and goodness of estimation.

By looking at Figure 3, this window will be composed of one closed dashed black line with the priors consisting of the previous estimate (or of the initial conditions for the first window) and by five closed dashed-dotted gray lines, where the indices of every factor change accordingly with the time step. Of course, the matrix A will be composed of six blocks following the rationale of Table 1, and vectors b and Δ grow accordingly.

## 4. Results

This section collects results obtained with the proposed approach and evaluated by means of real data acquired by an instrumented Ferrari 250 LM Berlinetta GT and made publicly available by Center for Automotive Research at Stanford [30]. A global navigation satellite system (GNSS)-aided inertial navigation system provides overall vehicle body motion, resulting in centimeter-level position accuracy and direct slip-angle measurement. The 2014 Targa Sixty-Six event served as the data collection venue that took place at the Palm Beach International Raceway, a 3.3 km-long track featuring 10 turns and a 1 km straight. Experiments have been carried out using an open-source implementation of the proposed framework (available online at https://github.com/MBDS/sideslip-angle-vehicle-estimation (accessed on 9 August 2021)).

**Figure 4:**
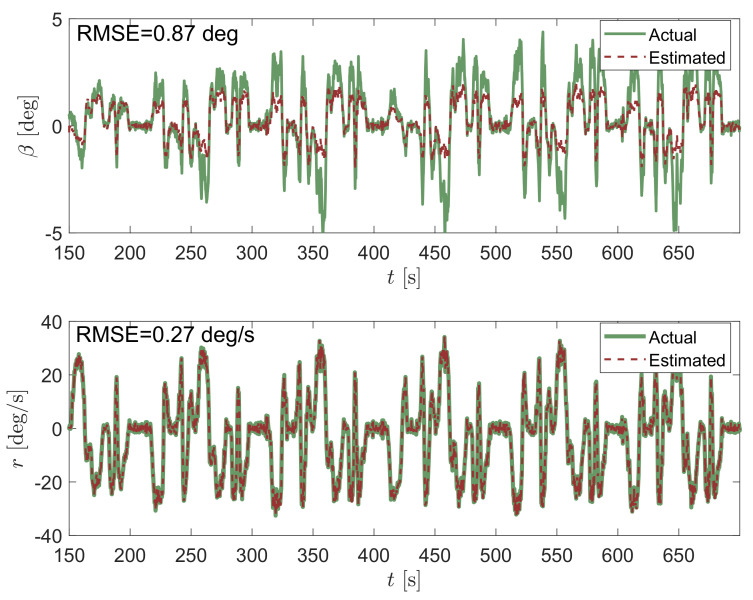
Vehicle sideslip angle β estimate at the top and yaw rate *r* at the bottom obtained by using a KF-based observer applied to the linear bicycle model.

For a thorough understanding of the advantages of the proposed method, the results obtained with the celebrated linear KF are first shown, which are well known in the automotive field. For the sake of brevity, the KF equations and the state-space form of the problem at hand is here omitted, since it is widespread in the literature (e.g., [13]). Figure 4 shows the estimation of the vehicle sideslip angle β and yaw rate *r* obtained via linear Kalman filtering during some laps of the Palm Beach International circuit. As expected, the unknown state associated to the yaw rate is estimated very well, since it is directly measured. In fact, only the sensor noise is filtered out, leading to an RMSE value of 0.27 deg/s. Note as well the relatively good estimation of β (RMSE of 0.87 deg) despite the approximation of assuming a linearized model. This is explained by the relatively small range of β values observed in practice and, in particular, for this race dataset.

**Figure 5:**
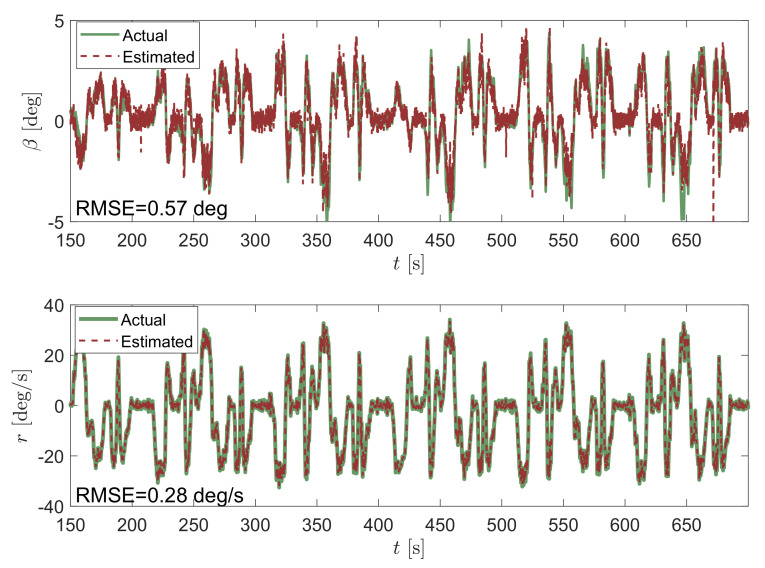
Vehicle sideslip angle β estimate at the top and yaw rate *r* at the bottom obtained by using FG applied to the linear bicycle model.

In Figure 5 the estimation of the sideslip angle β and yaw rate *r* is shown, but this time obtained from the FG-based observer. As seen from the figure, the estimator can track β more closely and even for higher values than KF, although it is based on a linear model. A corresponding RMSE of 0.57 deg is achieved that is a 34% improvement over the KF implementation. Note that the numerical estimators used for the FG are capable of optimally handling strongly nonlinear models due to their iterative nature, but in this particular case this advantage is not exploited due to the linearity of the model. In fact, the adopted nonlinear solver (Gauss–Newton) only ran a single iteration before detecting that it reached the optimum.

**Figure 6:**
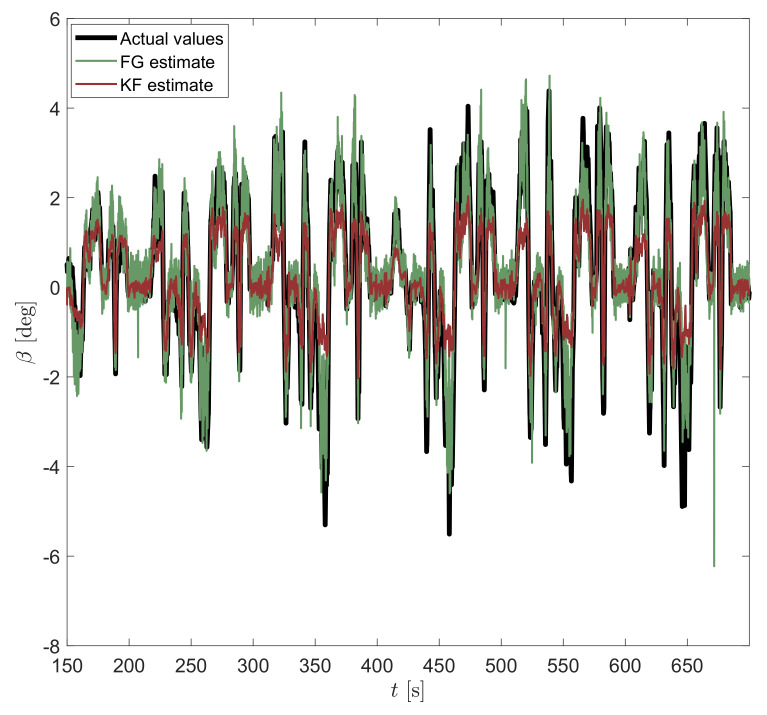
Estimation of vehicle sideslip angle β by considering both KF and FG estimators with a window of 5 samples.

**Figure 7:**
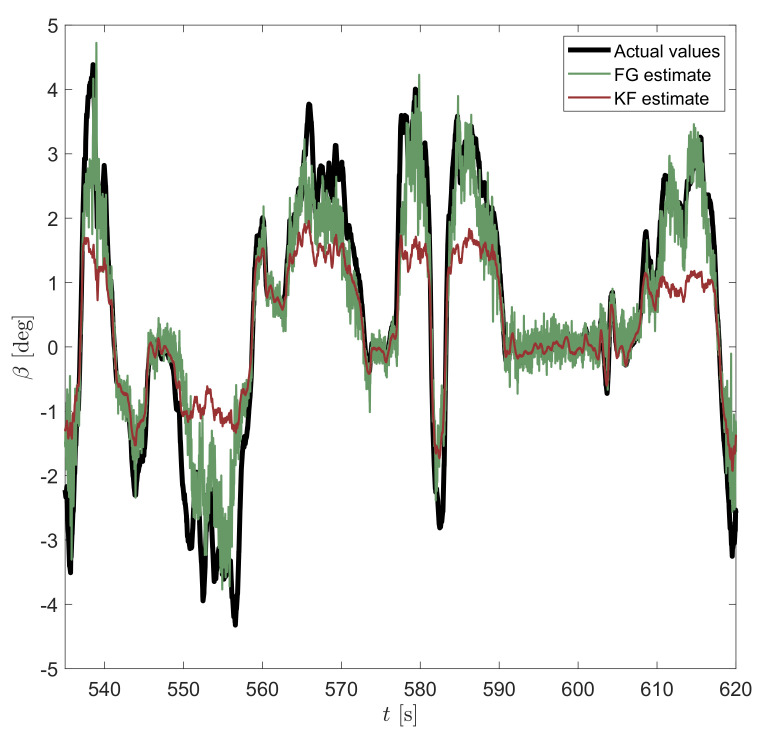
Estimation of vehicle sideslip angle β by considering both KF and FG estimators for small values of the sideslip angle.

On the other hand, one fundamental difference between the FG solver and the KF is the use of a sliding-window estimator for the former, which is then capable of smoothing out sensor noise much more effectively than KF is able to by sequentially processing time steps one by one. However, for very small β angles (see the whole comparison in Figure 6 and detailed view in Figure 7), KF seems to provide a less noisy output. This effect may be explained by the use of different tuning parameters in both approaches.

**Figure 8:**
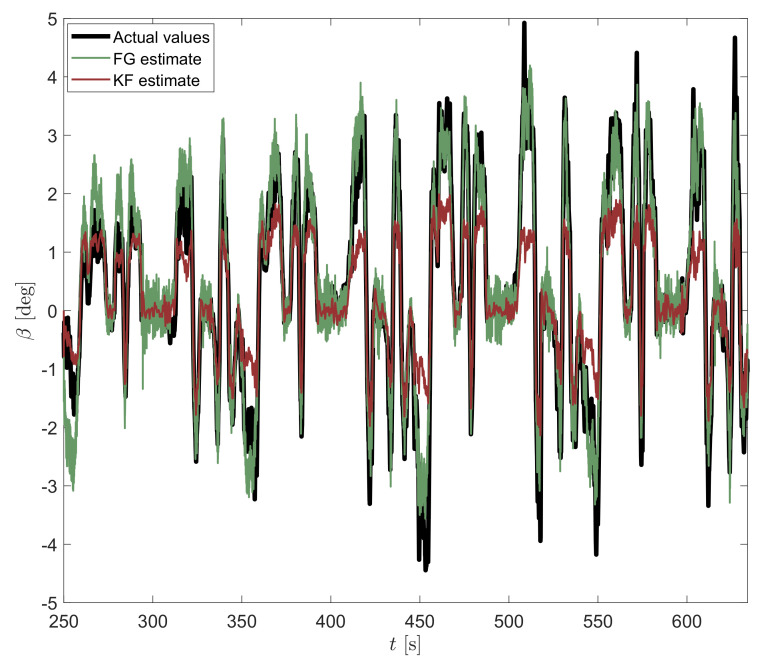
Estimation of vehicle sideslip angle β by considering both KF and FG estimators with a window of 5 samples, for another set of real data.

For completeness, another experiment performed on a different set of real data gathered in a different race session is displayed in Figure 8. Again, the KF shows the best performance in the linear region characterized by small β values, but fails for higher values of sideslip angle. In contrast, the FG is noisier for small β values but well estimates the actual sideslip angle for higher values, which are the most interesting for vehicle handling and passenger safety.

**Figure 9:**
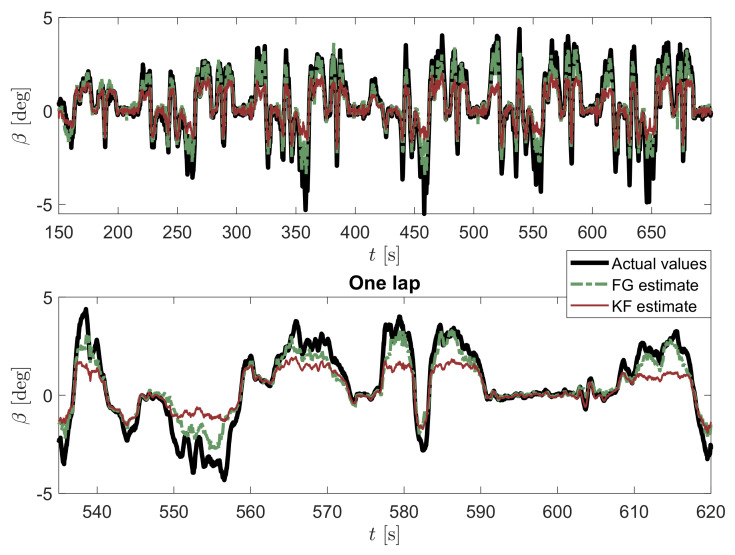
Estimation of vehicle sideslip angle β by considering both KF and FG batch estimators.

**Figure 10:**
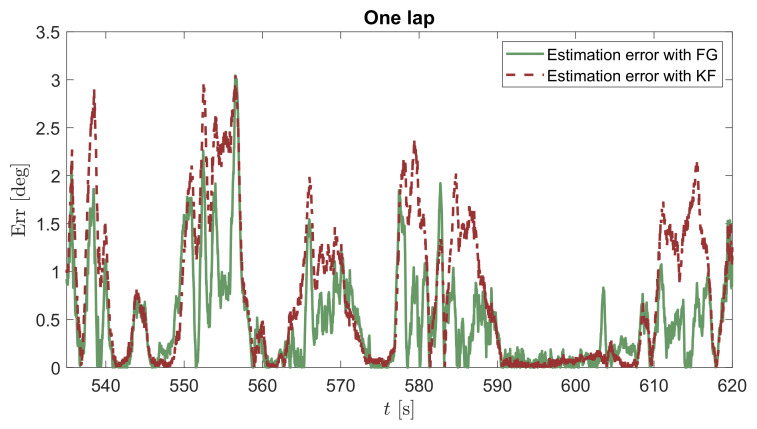
Absolute error in the estimation of sideslip angle β by considering KF against the FG batch estimator.

In order to show the potential of the proposed method to cope with large datasets by exploiting the variable sparsity, the batch estimation is reported in Figure 9 on the total number of samples acquired. As one can see, both the advantages of KF and FG with sliding window method are obtained, consisting of a good estimation for high values of β beyond the linear region and a very smooth estimate, especially in the linear one, similar to the KF, where a better visualization is achieved at the bottom of the figure in a shorter period of time, associated to a single lap. Moreover, Figure 10 displays the absolute error of the sideslip angle estimation performed with KF (red dashed line) and FG (green solid line) with respect to the ground truth, as further proof of the validity of the proposed approach. As can be seen, the error associated with the KF is almost always higher than the FG error.

**Figure 11:**
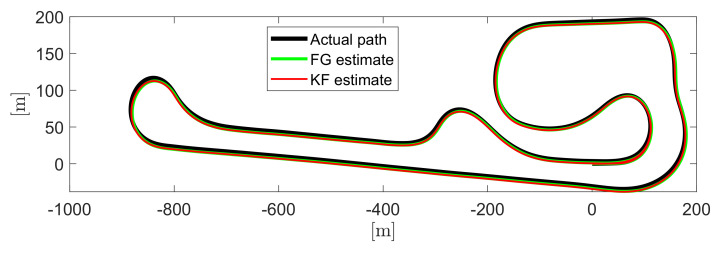
Path followed by the vehicle during a single lap: black is ground truth, red represents KF, and green indicates FG.

Finally, for the sake of completeness, the path followed by the vehicle during a single lap is shown in Figure 11, effectively obtaining a path shape that matches the Palm Beach International Raceway. The black path is the actual one obtainable from GPS data or by exploiting the actual yaw rate, sideslip angle, and longitudinal and lateral velocities provided by the dataset. As one can see from the figure, the green path representing the shape of the circuit estimated via FG is closer to the black path than is the red one corresponding to the path estimated by using KF for most of the route, especially for sudden curves, in agreement with the previous results.

## 5. Conclusions

In this paper an estimator grounded in the factor graph theory is applied for the first time to the estimation of the sideslip angle of a vehicle during motion. A linear single-track model is considered in this research as the vehicle model for the estimator. The performance of the proposed estimation approach is evaluated by using real data and contrasted with the performance obtained from the standard Kalman filter. It is demonstrated that the proposed method accurately estimates the sideslip angle even when its value exceeds the linearity range, which represents the more critical situation in terms of vehicle handling and passenger safety, and thus the knowledge of the correct β value becomes more important. On the other hand, for small values of the sideslip angle, the Kalman filtering preserves its superiority, as expected. Nonetheless, a batch estimation leads to a good estimate of the sideslip angle with a level of noise similar to that achieved using the KF. However, it is worth pointing out that higher values of the sideslip angle can be correctly estimated by keeping a linear bicycle model with very few parameters with respect to other more complex vehicle models, representing an important aspect in terms of practical implementation in real commercial vehicles.

Next steps will take into account FGs applied to more complex (nonlinear) vehicle models, in order to investigate the effectiveness and the ability to achieve even more accurate estimates, as well as other aspects related to vehicle dynamics that cannot be represented with the bicycle model.

## Figures and Tables

**Table 1:** Matrix A and vector b for the first three steps of estimation.

Factor	$\Delta_{1}$	$\Delta_{2}$	$\Delta_{3}$	$\Delta_{4}$	$\Delta_{5}$	$\Delta_{6}$	*b*
p1	$A_{1,1}$						$b_{1}$
p2		$A_{2,2}$					$b_{2}$
m1		$A_{3,2}$					$b_{3}$
m2	$A_{4,1}$	$A_{4,2}$					$b_{4}$
d1	$A_{5,1}$	$A_{5,2}$	$A_{5,3}$				$b_{5}$
d2	$A_{6,1}$	$A_{6,2}$		$A_{6,4}$			$b_{6}$
m3				$A_{7,4}$			$b_{7}$
m4			$A_{8,3}$	$A_{8,4}$			$b_{8}$
d3			$A_{9,3}$	$A_{9,4}$	$A_{9,5}$		$b_{9}$
d4			$A_{10,3}$	$A_{10,4}$		$A_{10,6}$	$b_{10}$
m5						$A_{11,6}$	$b_{11}$
m6					$A_{12,5}$	$A_{12,6}$	$b_{12}$

## Appendix A

In this Appendix, details about matrix A and vector b in Table 1 are provided for the convenience of the interested reader. Starting with matrix A, two different kinds of block matrix can be found: one associated to the first step with priors and one associated to the other steps with the dynamic model. By recalling that

(A1) $$Q^{-\frac{1}{2}}=\mathrm{diag}\left(\frac{1}{\sigma_{\beta }},\;\frac{1}{\sigma_{r}},\;\frac{1}{\sigma_{\dot{\varphi }}},\;\frac{1}{\sigma_{a_{y}}}\right)$$

the former kind of block matrix is a 4×2 matrix where:

(A2) $$\left[\begin{array}{ccc} A_{1,1} & \; & 0\\ 0 & \; & A_{2,2}\\ 0 & \; & A_{3,2}\\ A_{4,1} & \; & A_{4,2} \end{array}\right]=Q^{-\frac{1}{2}}\left[\begin{array}{ccc} 1 & \; & 0\\ 0 & \; & 1\\ 0 & \; & -1\\ \frac{C_{f}+C_{r}}{m} & \; & \frac{C_{f}l_{f}-C_{r}l_{r}}{mu_{k}} \end{array}\right]$$

The longitudinal velocity $u_{k}$ is that measured at the specific time step on which the priors are applied—for instance, for the first time step with priors associated to initial guess, $u_{k}=u\left(k=1\right)$. The other terms are constant in this work. The latter kind of block matrix is a 4×4 matrix obtained as in the following:

(A3) $$\left[\begin{array}{ccccccc} A_{1,1} & \; & A_{1,2} & \; & A_{1,3} & \; & 0\\ A_{2,1} & \; & A_{2,2} & \; & 0 & \; & A_{2,4}\\ 0 & \; & 0 & \; & 0 & \; & A_{3,4}\\ 0 & \; & 0 & \; & A_{4,3} & \; & A_{4,4} \end{array}\right]=Q^{-\frac{1}{2}}\;H_{k}$$

with $H_{k}$ depending on the *k*-th time step. A smoother with a window comprising 5 samples has an A matrix composed of one matrix (A2) and five matrices (A3) arranged on the diagonal.

Vector b grows accordingly with matrix A. By looking at Equation (18), the parentheses enclose the prediction error between the actual value and the estimated one. Therefore:

- For priors, the actual value is the guess and the function is the difference between these two values; hence:
  (A4) b1=β0−β0+β0/σβ
  and $b_{2}$ follows the same rationale.
- For dynamic factors, the actual value is zero, since it is the difference between the forward value and the same obtained by integrating the differential equation; for instance:
  (A5) $$b_{5}=\left[0-\beta_{1}+\left(1-dt\frac{C_{f}+C_{r}}{mu_{1}}\right)\beta_{0}-dt\left(\frac{C_{f}l_{f}-C_{r}l_{r}}{mu_{1}^{2}}+1\right)r_{0}+dt\frac{C_{f}\delta_{1}}{mu_{1}}\right]/\sigma_{r}$$
  and $b_{6}$ follows the same rationale.
- Finally, measures follow this rationale (only yaw rate is taken for the sake of brevity):
  (A6) $$b_{3}=\left({\dot{\varphi }}_{1}+r_{0}\right)/\sigma_{\dot{\varphi }}$$

where, naturally, all indices change according to the time step.
